# Association between the Timing of Pre-Workout Macronutrient Intake and Rated Appetite among Resistance-Trained Adults in Jbeil, Lebanon

**DOI:** 10.3390/ijerph20032399

**Published:** 2023-01-29

**Authors:** Lea Nasr, Yonna Sacre, Randa Attieh, Haider Mannan

**Affiliations:** 1 Faculty of Arts and Sciences, Department of Human Nutrition and Food Sciences, Holy Spirit University of Kaslik, Jounieh P.O. Box 446, Lebanon; 2 Translational Health Research Institute, School of Medicine, Western Sydney University, Campbelltown, NSW 2560, Australia

**Keywords:** appetite behavior, resistance training, nutrient timing, macronutrients, training intensities

## Abstract

Macronutrients play an important role in appetite regulation. In addition, adequate nutrient and energy intake, which may be altered by exercise-induced appetite fluctuations, is required to ensure important training outcomes. However, findings regarding appetite responses to macronutrient consumption before training and to different resistance training intensities remain inconclusive. This study investigated the association of three types of macronutrient intake before different intensities of resistance training with appetite. A purposive cross-sectional design was used to collect data from 280 resistance-trained individuals (mean age 26.4 ± 5.8 years) representing five gyms located in Jbeil, Lebanon, and who completed an online questionnaire. Data collected included socio-demographics, nutritional strategies followed by each respondent, training characteristics, and appetite rating before, during and after exercise using a validated visual analogue scale (VAS). A short-term suppression of appetite was reported during resistance-training, with no significant difference in exercise intensities (*p* > 0.05). In addition, low-fiber carbohydrate and protein food/beverage content consumed 30–60 min before training had an advantage in appetite suppression. In summary, these findings suggest that resistance training combined with pre-workout consumption of a whole meal was associated with appetite suppression, at least during the short period of exercise. From the perspective of appetite control and energy balance, the critical factor is the quantity and quality of macronutrient food sources, in addition to the timing surrounding training of nutrients ingested.

## 1. Background

Staying active can help in keeping the body healthy and reduce the risk of having health complications [1]. In addition, many studies have shown the importance of resistance training for the desire to eat [2], or what is called “appetite”, which is also associated with weight regulation and energy balance [3]. Furthermore, it has been reported, according to a study conducted by Poon et al. [4], that after a workout, people are not able to eat because of the suppression of their appetite [4], so they tend to skip the post-workout meal, which may alter muscle protein synthesis, recovery, and training adaptations. In this same study [4], higher blood lactate levels and appetite suppressor metabolites were observed immediately after HIIT and VICT. In addition, similar results were reported in a study conducted in 2021 by Freitas et al. [5] suggesting higher levels of blood lactate concentration compared with the baseline, which remained high 30 min after resistance training. Many factors influence appetite and the desire to eat among active people, including food composition and metabolism [6,7].

The association of macronutrients with appetite is controversial. Some studies suggest higher level of satiety response after protein and fat intake compared to carbohydrate [8,9] and others reported that carbohydrate intake is more satiating than fat [10]. In contrast, other research suggested no differences in appetite responses among macronutrients [11].

Little is known regarding the response of appetite to the timing and the type of macronutrients consumed. Some evidence suggested that the decrease in glucose levels stimulates the initiation of food intake [12]. The impact of protein on satiation has been hypothesized and proposed but not confirmed [13,14]. Protein intake stimulates the release of metabolic hormones and peptides from the gastrointestinal tract, providing an impact on appetite regulation in the brain [15]. Some research suggests that high protein diets can help increase satiety and reduce energy intake more than a consistent carbohydrate (CHO) and fat diet [15], and help lower hunger [16]. The association of fat with appetite has been studied since the 1900s, when Kennedy proposed the theory that food intake is controlled by a lipostatic mechanism associated with a circulating component, known as leptin [17]. In 2015, Marciani et al. [18] reported that dietary fat intake can lead to higher fullness responses due to its impact on the gastric volume compared to a high-carbohydrate meal. Yet, the same study suggested that a high-fat low-carbohydrate diet should not be followed to control short-term food intake since high-fat food has higher energy density. As a result, food having lower fat would be needed in order to satisfy individuals’ energy requirements to prevent excess fat deposition and obesity.

The majority of studies focused only on the effect of the intensity of endurance training on appetite [4,19,20,21,22] and only a few studied the regulation of appetite induced by resistance training [2,23,24]. On the other hand, no studies have investigated the association between macronutrient intake before both resistance and endurance training, and appetite post-training.

To our knowledge, no studies have been conducted and/or published on the association between macronutrient intake before resistance workouts and appetite responses in the Lebanese population, although these are important factors to control weight management, encourage exercising, and maintain a healthy lifestyle. The aim of this study was to assess, on one hand, the rate of appetite after consumption of carbohydrate, fat, and protein 30–60 min before different exercise intensities and, on the other hand, the gender-related appetite responses after resistance training.

## 2. Material and Methods

### 2.1. Subjects

The Research Ethics Committee (REC) of the Higher Center of Research (HCR) at Holy Spirit University of Kaslik (USEK) approved the study protocol. Two hundred and eighty (151 males, 129 females) healthy resistance-trained adults aged 20 years and above agreed to participate. Subjects were performing resistance and/or endurance exercises (179 resistance, 21 endurance, 141 both) at five selected gyms located in the city of Jbeil- Lebanon, and did not comprise pregnant or menopausal women, had no thyroid disease, and did not take any hormonal and/or appetite suppressive medications. The physical characteristics of the subjects were: mean age 26.4 ± 5.8 years; body mass index (BMI) 24.1 ± 3.9 kg/m^2^. Subjects had different physical activity levels, which depended on their daily activities and the period of training experience: 50 were beginners, training the entire body two to three nonconsecutive days per week; 109 were intermediate, exercising three to five days per week with two or more days in a row of training; 57 were advanced, training four to six days per week performing 2 sessions per day (8–12 sessions per week) with three consecutive training days; and 64 were certified professional trainers [25].

### 2.2. Study Design

The research study had a cross-sectional design in which data were collected from resistance training practitioners in Jbeil for four months (August 2021–December 2021). The association between macronutrients consumed before training, exercise intensity, and gender, and appetite responses after training, was analyzed to understand how different factors could affect appetite responses in active people.

### 2.3. Procedure

Because of the current situation of COVID-19 and the economic crisis, the number of health and fitness clubs in Lebanon may have varied, and, to the authors’ knowledge, there is no evidence concerning the current number of these clubs as many may have closed. Therefore, this research was limited to sports practitioners performing in gym clubs located in the city of Jbeil, Lebanon. The selection of gyms followed the objectives and participant characteristics needed for this study. Since this study targeted resistance training practitioners for individuals aged 20 years and above, gyms with the availability of weightlifting and CrossFit equipment for the mentioned age groups were recruited. Out of a total number of 26 gym clubs located in Jbeil, only 11 were able to meet the study requirements. From the 11 gyms available, 5 agreed to participate in the present study. Owners of these gyms were contacted via social media (e.g., WhatsApp, Instagram, Facebook) or visited at their gyms and asked about the average number of gym members.

Some gym owners allowed visits to their gyms to ask their members in the weightlifting area and in the group sessions to participate in the study. The individuals who agreed to participate in the study were asked to complete the questionnaire sent to them as an URL address via Instagram, Facebook, or WhatsApp. The survey included 64 questions, distributed into seven sections presented in English, using a Google Forms program, and completed in 5–10 min. Four sections were adapted from the Weight and Lifestyle Management Questionnaire [26], including sociodemographic information, anthropometric measures, medical history, and physical activity, in addition to three sections for appetite rating: before, during, and after training. All questions were asked in English.

A purposive sampling method was used to concentrate on participants with characteristics meeting the objectives of the present research study requirements. Two hundred and eighty sports practitioners, from gyms selected for this, study included resistance trainers, with an age above 20 years. Participants were divided into two groups depending on their gender and the intensity of the exercise performed.

### 2.4. Measurements

#### Exercise Intensity Analyses

As a general rule, the intensity of the exercise is characterized by the percentage of the maximum heart rate (MHR). Lower resistance exercises’ repetitions and a longer rest time between sets provide temporal elevation in blood pressure [27].

The intensity of training was assessed through the participants’ current repetitions of the exercise performed. Data were collected from the Physical Activity section in the questionnaire, in which participants indicated their current exercises’ repetition strategy from the four options, including: single-repetition event (1–2 repetitions) and multiple-repetition event (3–5 repetitions) considered as high-intensity training protocols. On the other hand, options for 6–12 repetitions and more than 12 repetitions were considered as moderate- to low-intensity training protocols.

### 2.5. Dietary and Pre-Workout Nutrient Intake Assessment

Dietary and food intake data were collected from the pre-workout food record section in the questionnaire. Participants estimated the portion of the selected 18 food and 7 beverage types, mostly consumed by the Lebanese population, and belonging to the different macronutrient groups consumed pre-workout according to the handy guide to serving size [28]. In addition, they selected the timing of the food consumed pre-workout from the following options: less than 30 min, 30–45 min, 1–2 h, 2–4 h, or more than 4 h. The pre-workout dietary record helped in studying the association between the timing of the different nutrients’ intake before training and appetite responses after training.

### 2.6. Appetite Analysis

Participants recorded their appetite rate depending on four questions: desire to eat, hunger sensation, fullness sensation, and ability to eat. Appetite was estimated by self-appetite rating records on the validated visual analogue scale (VAS) [4], before, during, and after training, graded from 1 to 10. The average appetite score was calculated using Poon et al.’s [4] formula, as shown below.

(1) How strong is your desire to eat?
(2) How hungry do you feel?
(3) How full do you feel?
(4) How much food do you think you could eat?

$$Appetite\;score=\frac{\left(desire\;to\;eat+hunger+\left(100-fullness\right)+prospective\;consumption\right)}{4}$$

The average score obtained was compared among the three different periods surrounding the training performance (before, during, and after), in order to assess the changes in appetite responses.

### 2.7. Statistical Analyses

Data are presented as mean ± SE. The one-way ANOVA test followed by a post-hoc test was conducted to examine the association of the timing of macronutrient type intake on appetite responses, and to determine whether there are any statistically significant differences between the impact of low-, moderate-, and high-intensity exercises on appetite.

A series of two-way ANOVA tests followed by a post-hoc test was conducted to investigate the following:

(1) Association between exercise intensity, the timing of macronutrient type intake, and appetite responses;
(2) Association between participants’ gender, the timing of macronutrient type intake, and appetite;
(3) Association between participants’ gender, age, and appetite responses.

Furthermore, an independent t-test was used to analyze the association between appetite rate after training with gender differences.

The significance level was set at 0.05. All statistical analyses were performed in IBM SPSS software version 26 for Windows.

## 3. Results

A total of 340 participants were enrolled in this study. Of those, 60 were excluded because of their endurance training practices, having a thyroid disorder or using medication treating a hormonal disorder, or living abroad or in other countries. None of the participants were using doping substances, pregnant or menopausal, or taking medications for weight loss or appetite reduction. As a result, 280 participants met the requirements of this research project.

### 3.1. Exercise Intensity Analyses

**Figure 1:**
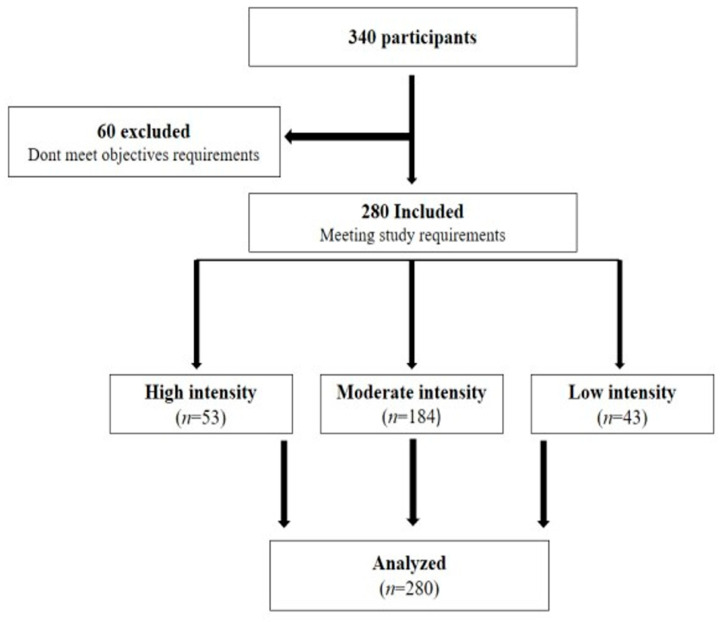
Participation flowchart depending on the intensity of training performed.

Since this study aimed to investigate appetite rating depending on different training intensities, participants were asked about their exercise repetition strategies to determine its intensity. From the 280 selected participants, 53 practiced high-intensity training, 184 moderate-intensity training, and 43 low-intensity training, as shown in Figure 1.

### 3.2. Dietary and Pre-Workout Nutrient Intake Assessment

**Figure 2:**
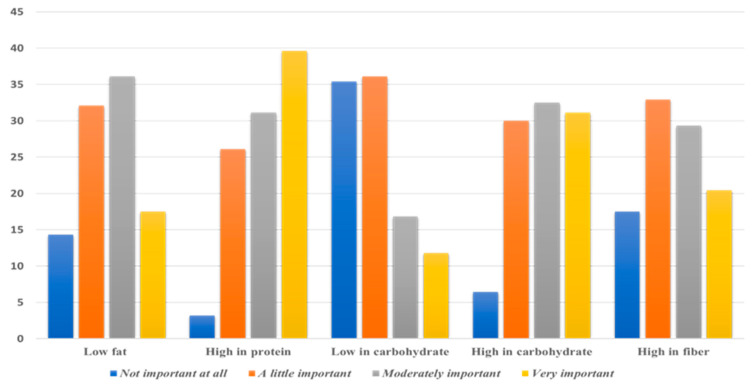
Preferred food type selection as before a workout (in percent).

Some participants preferred working out in a fast state. Statistics of this study showed that 221 (78.9%) participants usually eat before training. On the other hand, 53.6% of the participants usually consider the intake timing of food and/or beverages before training, while 37.1% sometimes consider the timing. Statistics showed that participants usually choose to eat low-fat (36.1%), high-protein (39.6%), and high-carbohydrate (32.5%) meals or snacks. On the other hand, the fiber content of meals or snacks showed little importance among participants (32.9%) (Figure 2).

### 3.3. Appetite Analyses

**Figure 3:**
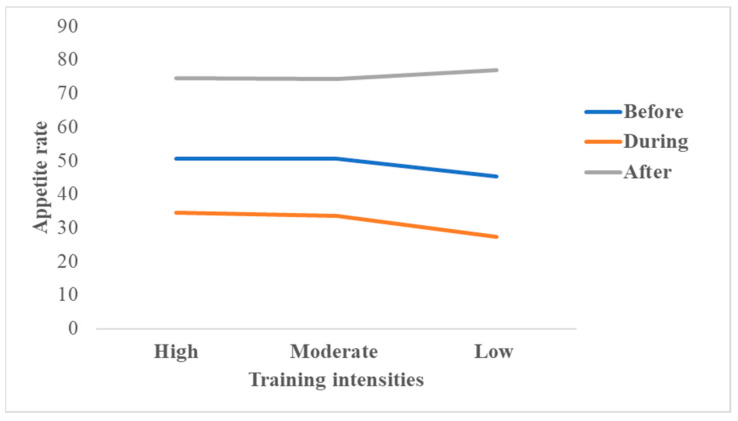
Appetite scores after training depending on the three training intensities.

A suppression of appetite was observed during the three resistance training intensities (*p =* 0.04). Then, it increased after, especially for low-intensity training (mean = 77.1 ± 0.73) compared to high- (mean = 74.5 ± 0.85), and moderate- (mean = 74.3 ± 0.80) intensity training. This increase was not significant (*p* > 0.05) (Figure 3).

**Figure 4:**
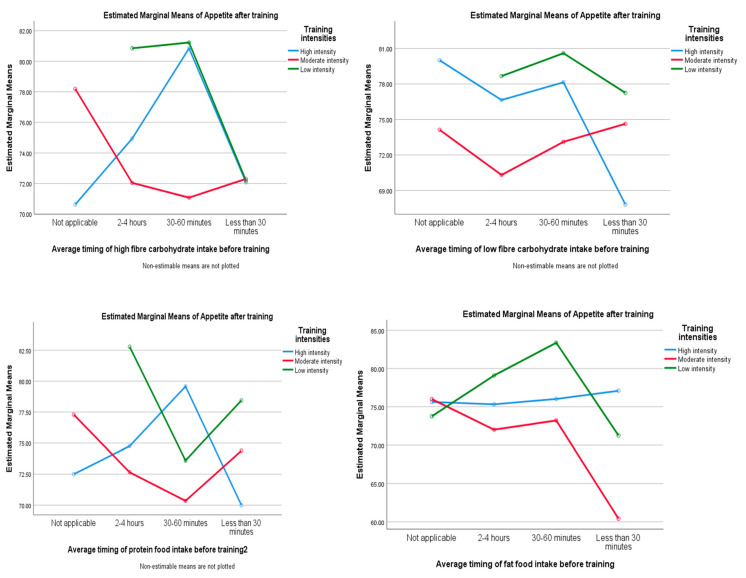
The mean of appetite depending on the timing of nutrient intake and training intensities.

A suppression of appetite was also observed after the consumption of carbohydrate, protein, and fat, especially 30–60 min before different resistance training intensities; however, this suppression was not statistically significant (*p* > 0.05) (Figure 4).

**Figure 5:**
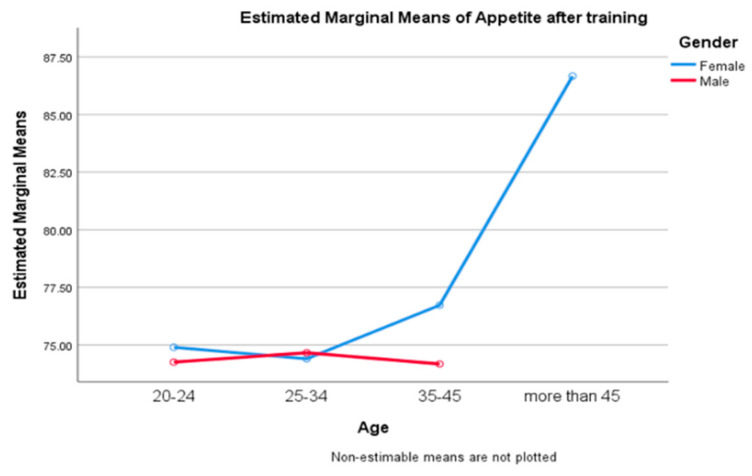
Appetite response depending on age and gender differences.

As mentioned previously, the appetite score was calculated depending on four sub-groups: desire to eat, hunger, fullness sensation, and ability to eat. In a further analysis (Figure 5), the desire to eat (*p* < 0.01), ability to eat (*p* = 0.032), and hunger sensation (*p =* 0.410) decreased during high-resistance exercise, compared with baseline. On the other hand, fullness sensation remained the same. This suppression was temporary, and it increased in the mentioned sub-scores after training compared to the rates recorded during training (Table 1).

There was no statistically significant association between age and appetite responses after training in men versus women (*p* = 0.933), according to the one-way ANOVA test (Figure 5).

## 4. Discussion

This was the first study investigating the effect of resistance training on appetite responses that took into consideration the timing of nutrients consumed before training in a cross-sectional study design. The findings suggest an association between macronutrient intake before higher training intensities with the desire to eat, ability to eat, and hunger sensation. Participants reported a suppression of appetite after consumption of food/beverages having high carbohydrate and protein contents, at least during the short period of high-intensity resistance training.

The benefits of physical activity in general, and resistance training specifically, have been reviewed considerably in recent years regarding weight management [1,29], in addition to health and cognitive benefits [24,30,31]. Some evidence showed that appetite responses can be regulated by physical training and that this can help with weight [4,5,21,23,32,33]. On the other hand, the study carried out by Dos Prazeres et al. [2] reported no alteration in hunger sensation among resistance training practitioners. This can be demonstrated by the fact that increased physical activity levels play a role in increasing energy expenditure during exercise, which should be restored, depending on the needs of each individual, for training recovery, adaptation, and hypertrophy. This energy and nutrient replenishment requires the ability to eat and an appetite, especially for high energy needs.

In the current study, results showed a suppression of hunger and desire to eat during training. The utilization of glucose as a primary source of energy, and the limited capacity of glucose storage in the liver [12], may explain this finding. It has been shown that, as exercise intensity increases and the duration is greater than 1 h, the utilization of ATP and CHO increases, as muscle glycogen becomes the main source of energy [4,34,35].

On the other hand, fats and especially proteins have limited oxidation levels during exercise, as reported in the study conducted by Spillane et al. [36]. This mechanism may contribute to relieving the feeling of hunger and reducing appetite sensations.

About 33% of the participants ascribed little importance to choosing high-fiber food before training. The consumption of low-fiber carbohydrate food from less than 30 to 60 min before training showed a suppression of appetite in high training intensities; this indicates that, at higher training intensities, the body relies on carbohydrate, however, at lower intensities, it relies on fat. These findings were supported by Loon et al. [37]. About 40% of the participants reported the high importance of protein consumption before training. However, protein alone was not significantly associated with appetite suppression, but it was when consumed with carbohydrate 30–60 min before training.

The present study also revealed that participants who consumed carbohydrate and protein 30–60 min before training showed a decrease in appetite rate. The results showed that this suppression of appetite was dependent on the training intensity as it was more significant after higher training intensities. These results confirm the association between high training intensity and appetite suppression reported in previous studies. A study conducted in Sao Paulo University (UNESP) by Freitas et al. [5] reported a decrease in hunger sensation among resistance-trained athletes after consumption of a balanced meal (15–20% protein, 50–60% carbohydrate, 25–30% fat) before 30–60 min of high-intensity resistance training. These results were also supported by a review study conducted by Dorling et al. [38], suggesting that higher levels of physical activity may provide improvements in the sensitivity of the appetite control system. However, this same review suggested further research is needed concerning the association between appetite response and exercise intensities. High-intensity training induced suppression of appetite in Rahmani-Nia et al.’s study in 2015, in addition to increased anorexigenic hormones (PYY and insulin) [23]. However, physical activity, independently of intensity, showed a significant association with the ability to eat and the desire to eat for a short period. These results support the findings of the study conducted in China by Poon et al. [4], which suggested that, despite the differences in lactate concentrations, appetite responses were similar among high-intensity interval training (HIIT), vigorous-intensity continuous training (VICT), and moderate-intensity continuous training (MICT).

The study’s results showed that appetite suppression was dependent on gender differences, since this suppression was slightly greater among men compared to women. This finding was contradicted by a review in England conducted by Thackray et al. [39], which suggested no differences in appetite rate after training between men and women. This might be due to differences in the study type, methodology, sampling method, and appetite perception, since appetite-related hormones were tested to analyze appetite responses among participants. Wade and Jones [40] reported that few studies are available investigating appetite in women compared to men due to the menstrual cycle, suggesting a decrease in energy intake during the follicular phase that causes appetite fluctuations.

In fact, the study found that appetite response was not directly and significantly associated with gender differences due to the menstrual cycle among women. Appetite was not directly associated with training intensity since not only did a high level of training induce appetite suppression, but also moderate training. However, our results showed that a pre-workout meal/snack containing carbohydrate and protein is optimal for satisfying the energy needed for the workout and to maintain appetite sensation after a short period.; then, appetite may increase later to compensate for energy used during exercise.

Despite the interesting data that can complement the scientific literature, this study had some limitations in the context of data collection and methodological procedure. The most relevant of these was the absence, due to cost limits, of an appetite-related hormone blood test, which would enable better analysis and appetite interpretation. In addition, appetite was only recorded after training in general, rather than comparing scores after, for example, 30 min, 1 h, or 2 h, in order to indicate the duration of appetite reduction before it increased later, and to understand the mechanism by which energy intake has increased after physical activity due to its effect on increasing energy expenditure.

Each nutrient is an important component in improving physiological training adaptations and preventing muscle and strength loss deprivation, and each macronutrient has an essential role in the diet of active individuals [41]. Nonetheless, from the perspective of appetite control and energy balance, the critical factor is the quantity and quality of macronutrients’ food sources, in addition to the timing surrounding training of the nutrients ingested [42].

## 5. Conclusions

It is important to conduct long-term experimental trials to better understand the association among and impact of nutrients consumed before different resistance training intensities on appetite rate, and, therefore, provide adequate nutritional strategies for resistance trained practitioners for better performance and energy intake. Further research is required to determine the duration of this appetite suppression and describe the desire to eat and fullness sensation of the participants in different time periods after a single training session, in addition to other lifestyle factors, including sleep management, that affect appetite responses among sports practitioners.

In summary, the findings showed that consumption of a complete whole meal containing carbohydrate and protein 2–4 h before training, and consumption of a small snack containing easy-to-digest carbohydrates and protein sources 30–60 min before training, can promote appetite stabilization and provide the energy required for resistance training, at least for the short-term. The suppression of appetite was observed in both men and women, but it was more significant among women. However, appetite responses among women may be inaccurate since the menstrual cycle and hormone changes can cause appetite fluctuations. Considering nutrient ingestion before training, as a key component in appetite regulation, can help athletes to control energy intake post-workout, depending on the individuals’ goals, by decreasing or promoting satiation.

## Figures and Tables

**Table 1:** Mean ± SE for appetite score before, during, and after training.

	Before	During	After
Desire to eat	5.07 ± 0.15	2.66 ± 0.13	8.44 ± 0.10
Ability to eat	5.33 ± 0.14	2.99 ± 0.13	8.30 ± 0.11
Hunger sensation	4.86 ± 0.14	3.10 ± 0.14	8.43 ± 0.11
Fullness sensation	5.15 ± 0.14	5.23 ± 0.16	5.20 ± 0.19

## Data Availability

Data is unavailable due to privacy or ethical restrictions.
